# Temperature based maximum power point tracking for photovoltaic modules

**DOI:** 10.1038/s41598-020-69365-5

**Published:** 2020-07-27

**Authors:** Josean Ramos-Hernanz, Irantzu Uriarte, Jose Manuel Lopez-Guede, Unai Fernandez-Gamiz, Amaia Mesanza, Ekaitz Zulueta

**Affiliations:** University of the Basque Country, UPV/EHU, Vitoria-Gasteiz, Spain

**Keywords:** Renewable energy, Electrical and electronic engineering

## Abstract

In this article authors propose a temperature based Maximum Power Point Tracking algorithm (MPPT). Authors show that there is an optimal current vs maximum power curve that depends on photovoltaic (PV) module temperature. Therefore, the maximum power point (MPP) can be achieved in very few commutation steps if the control forces the PV module to work in temperature dependent optimal curve. Authors shows how this PV module temperature based MPPT is stable and converges to MPP for each temperature. In order to proof its stability, authors propose a Lyapunov energy function. This Lyapunov energy function has positive values for all values except into MPP given the PV module temperature. This Lyapunov energy function has negative increment along each time step. Hence, the stability of temperature based MPPT can be demonstrated. The proposed MPPT algorithm proposes a current set point. This current set point is obtained with instantaneous PV module power and temperature dependent maximum power vs optimal current curve. Stability is analysed for different temperature levels. Optimal current vs maximum power curve has been modelled by a line. The lines’ coefficients depend on PV module temperature. Proposed Lyapunov energy function is not symmetric about equilibrium or MPP because MPPT algorithm and PV module dynamic have no symmetric behaviour about this equilibrium point.

## Introduction

Photovoltaic market shows a steady and continuous growth worldwide. This advance is possible thanks to the large reduction of the cost and to the efficiency increase of commercial photovoltaic modules. A growth around 20% has been achieved in the recent years in the world photovoltaic market. A descending trend in the price of the modules and other basic components of the photovoltaic facilities has been observed since 2010. In this context, photovoltaic energy has become a feasible alternative to deal with the growing request of energy.

An efficient MPPT algorithm is a key issue to improve the energy incomes of photovoltaic panels in addition to increase overall system efficiency. Its fundamental advantage based on neural networks for modelling is that no prior knowledge of the physical parameters related to the PV system is required^1^. Lopez-Guede et al.^2^ presented a neural networks based model of a PV module Mitsubishi Electric PV-TD185MF5 (185 Wp) which is located on the roof of the Faculty of Engineering of Vitoria-Gasteiz (University of the Basque Country, Spain). A data set of 63,000 samples collected during 18 months (from August 2013 to February 2015) has been used. That is the model used in present work.

Nowadays, several methods are being used to get the maximum energy from the solar cells^3^. Dang et al.^4^ increased the efficiency of the nanowire CdS/CdTe solar cell from 9 to 11% by using a 10 nm thick molybdenum oxide as a transparent layer. On the other hand, single axis and dual axis solar trackers are also used to enhance the solar insolation collection capability by orienting the solar^5^. While various Maximum Power Point Tracking (MPPT) algorithms such as Incremental Conductance (IC) algorithm^5–7^, Perturb and Observe (P&O)^8–10^, Artificial Neural Network (ANN)^10^, fuzzy logic^11^ , and Particle Swarm Optimization (PSO)^12^. A review of the MPPT based on PV panel and power converter characteristics can be found in Motahhir et al.^13^.

The P&O algorithm requires a reduced implementation cost with good performance. This algorithm can effectively address stable variations around the MPP and dynamic variations of the environmental conditions such as temperature and solar radiation^14^. Likewise, Alik et al.^15^ uses this algorithm because they also consider that the main advantages of this method are its low cost, simplicity and precision. To improve the performance of the installation, Moshksar et al.^16^ proposed a new MPPT algorithm comparing it with several versions of P&O. In the case of Ramos-Hernanz et al.^17^, they compare the behaviour of three versions of the algorithm P&O in simulation and in real life.

Other authors use the IC MPPT control algorithm, because it measures precisely the temperature of the PV module and tracks the MPP without oscillations^5^. In the opinion of Chen et al.^18^, the IC control algorithm improves the behaviour of the P&O. In the case of Radjai et al.^19^ they develop a new IC controller based on fuzzy logic with direct control to eliminate all the disadvantages of the classic control algorithm IC. In Ramos-Hernanz et al.^20^ the behaviour of the IC algorithm in simulation and in a real installation is compared. In the work of Rezk et al.^21^ four of these MPPT are compared: P&O, IC, Hill Climbing (HC) and Fuzzy Logic Controller (FLC). In the case of Cortajarena et al.^22^ the algorithms used are HC, P&O and a new Sliding Mode Control (SMC) algorithm.

A new MPPT based technique that combines simplicity and effectiveness was proposed by Chaieb et al.^23^. The proposed method combines the Simplified Accelerated Particle Swarm Optimization (SAPSO), which is a variant of the Particle Swarm Optimization (PSO) algorithm and the classic HC algorithm.

According to Li^24^, the P&O and IC methods are the most used. The P&O method can work well when solar irradiance and temperature do not change rapidly over time. Low cost P&O based controller can be implemented with a simple analogue circuit or microcontroller. But this method has the disadvantage that it cannot track the MPP quickly and the output power oscillates around the MPP. The method IC has a better performance than the algorithm P&O but its implementation is more difficult. In Bayod-Rujula et al.^25^, researchers ensure that control algorithms P&O and IC are the most used due to their simplicity. However, these algorithms have some weaknesses that affect their efficiency, especially when there are rapid changes in irradiance or partial shading of the installation. In order to solve some of the problems presented by the algorithm P&O, Li et al.^26^ proposed an MPPT control strategy with Variable Climate Parameters (VWP), which has the ability to track the MPP more quickly and compares this new algorithm to the P&O algorithms and with the fuzzy control method. However, Jamal et al.^27^ compared a new control algorithm based on Finite Time Sliding Mode Control (FTSMC) with the P&O methods and the standard SMC algorithm.

One of the main problems in PV installations is the control of the MPPT when there are partial shading conditions. Mohapatra et al.^28^ perform a very thorough review of all modern MPPT algorithms that are used in PV installations, reaching the conclusion that the choice of MPPT control algorithm depends on the application, hardware availability, cost, convergence time, accuracy and reliability of the system. In the study of Hadji et al.^29^, a control algorithm based on Genetic Algorithms is compared to the standard P&O and IC. Unlike Yatimi et al.^30^, that does this comparison with the control algorithms P&O and SMC, and Ramos-Hernanz et al.^31^ compares P&O, IC and SMC. A new control algorithm based on SMC was also presented in Ramos-Hernanz et al.^32^.In this study, the performance of the algorithm was simulated and compared with real tests. The results of the simulations showed good agreement with the experimental ones.

Another possible MPPT technique family are fuzzy logic based controllers^33,34^. These studies use fuzzy logic based controllers with time delay voltages and currents. The relationship between different variables is done with fuzzy logic rules, being set or trained with real data.

Shahid et al.^5^ proposed an Incremental Conductance based algorithm for indoor PV system under controlled temperature conditions. An intermediate optical stage called temperature controller was introduced in the study to transport the temperature of the concentrated light and hereafter solar PV panel to Standard Test Conditions (STC).A MPPT based on variable step sized incremental conductance algorithm was inserted at load side to ensure the conditioned and better quality power at the output.

The highest of the power-voltage curve of the PV panel is usually expressed as MPP, which is identified by a search algorithm. Lately, multipurpose Maximum Power Point Tracking (MPPT) algorithms have been suggested and implemented for outdoor solar PV systems^35–38^. Yadav et al.^39^ and Zahedi^40^ studied the effect of temperature on MPP of the solar panel and stated a reduction in maximum output power with increasing temperature. As the temperature of the PV material increases, the band gap of the semiconductor material is reduced giving more energy to the electrons. The lower band gap increases the carrier and therefore, the mobility of the carriers is reduced. They recombine before reaching the other electrode. The more the recombination the more saturation current. Thakur et al.^41^ investigated the need of open circuit voltage on temperature of the solar cell. Yadav et al.^42^ demonstrated that in low concentration PV systems, temperature is increased with high concentration of light and need an optic solution to balance the temperature effects. A detailed study about the effect of temperature on solar cell’s open circuit voltage and the MPP for diverse temperatures was presented in Yadav et al.^42^.

In the current study, authors propose a new MPPT that uses only the PV temperature. An optimal current *vs* power curve can be defined for each temperature. This optimal curve is applied in order to achieve the MPP. In “Problem statement”, authors describe a PV system and its dynamics; the maximum power points are also calculated in this section. This analysis emphasizes the dependence between optimal current and temperature. In Section *Temperature based MPPT algorithm*, authors propose a control law that profits the dependence of maximum power and optimal current for a given temperature. In Section *Temperature based MPPT algorithm stability analysis*, the stability of the complete system is demonstrated by means of a proposed Lyapunov energy function. Finally, a section is dedicated to explain the conclusions of the present study.

## Problem statement

### Photovoltaic module model

The most import stage in temperature based MPPT stability analysis is the PV electrical behavior characteristics. In order to identify this electrical behavior, authors propose several measurements.

**Figure 1:**
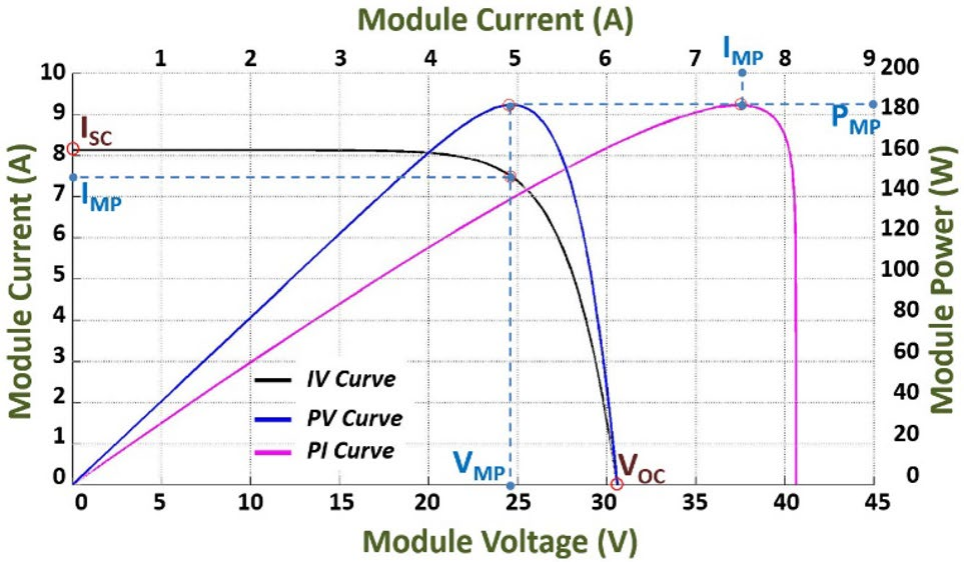
Mitsubishi Electric PV-TD185MF5 (185 Wp) PV module electrical characteristic.

By varying the load connected to the PV modules, and keeping the temperature and irradiance constant, the current and voltage change, producing the characteristic curves of the PV modules, as shown in Fig. 1. Conceptually, the curve represents the combinations of current, power and voltage in which the PV module could operate.

The equivalent electrical circuit of a solar cell consists of a current source (*I*_*L*_) dependent on irradiance. That irradiance is the current photo generated for a fixed value of solar radiation, a diode (*D*), a parallel resistance (*R*_*SH*_) which is related to the imperfections of the union p-n and represents the leakage current and a series resistance (*R*_*S*_). *R*_*S*_ represents the internal resistance of the material to the current flow and is associated with different effects such as the resistance of the contacts, of the semiconductors themselves and of the metallic diodes that form the frontal metallization mesh.

The mathematical model of the solar cell is obtained from its electrical representation and depends mainly on the solar radiation and the operating temperature of the cell.

**Figure 2:**
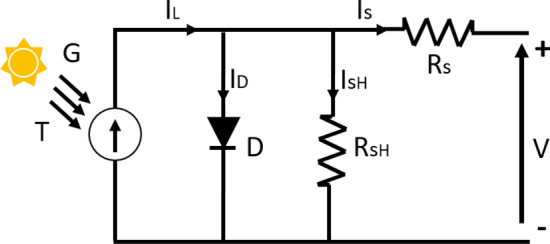
Equivalent circuit of a PV module.

By applying Kirchhoff to the circuit illustrated in Fig. 2, the current generated by the PV module is determined by Eqs. (1) and (2):

1 $$ I = I_{L} - I_{D} - I_{SH} $$

2 $$ I = \overbrace {{I_{L} }}^{{I_{L} }} - \overbrace {{I_{0} \left( {e^{{\frac{{q\left( {V + IR_{S} } \right)}}{{aKT_{C} }}}} - 1} \right)}}^{{I_{D} }} - \overbrace {{\frac{{V + IR_{S} }}{{R_{SH} }}}}^{{I_{SH} }} $$

where *I* is the output current of the solar cell, *I*_*L*_ is the photo generated current, *I*_*D*_ is the current through the diode, *I*_*SH*_ is the resistance loss current in parallel. *I*_*0*_ is the inverse saturation current of the diode, *q* is the charge of the electron (1.6029 × 10^–19^ C), *V* is the voltage generated in the solar cell, *K* is the Boltzmann constant (1.3819 × 10^–23^ J/K), *T*_*C*_ is the operating temperature of the solar cell and *a* is the ideality factor.

The photo generated current varies depending on the solar radiation and the operating temperature of the solar cell, as described in Eq. (3):

3 $$ I_{L} = \frac{G}{{G_{Ref} }}\left[ {I_{{L_{Ref} }} + \alpha_{{I_{SC} }} \left( {T_{C} - T_{{C_{Ref} }} } \right)} \right] $$

where *G* is the solar radiation at given conditions (W/m^2^), *G*_*Ref*_ is the solar radiation at standard conditions (1,000 W/m^2^), *I*_*LRef*_ is the current photo generating at reference conditions, which takes the value of the short-circuit current (*I*_*SC*_). α_ISC_ is the short-circuit current temperature coefficient and *T*_*CRef*_ (298°K) is the working temperature of the solar cell at standard conditions (298°K).

The saturation current is responsible for some solar panel inefficiencies. Depending on the temperature it will increase as the solar cell temperature increases, lowering the efficiency, as is described in Eq. (4):

4 $$ I_{0} = I_{{0_{Ref} }} \left( {\frac{{T_{C} }}{{T_{{C_{Ref} }} }}} \right)^{3} e^{{\left[ {\frac{{qE_{G} }}{aK}\left( {\frac{1}{{T_{{C_{Ref} }} }} - \frac{1}{{T_{C} }}} \right)} \right]}} $$

where *I*_*oRef*_ is the inverse saturation current at reference conditions and *E*_*G*_ is the band gap energy.

This inverse saturation current (*I*_*oRef*_) corresponds to the leakage of load carriers through the junction p-n, as a result of their recombination in a neutral zone of the semiconductor. It is calculated by Eq. (5):

5 $$ I_{{0_{Ref} }} = \frac{{I_{SC} }}{{\left( {e^{{\frac{{q\left( {V_{OC} } \right)}}{{N_{S} aKT_{C} }}}} - 1} \right)}} $$

Usually, the power produced by solar cells is low. Therefore, it is necessary to group them in series or parallel to achieve the desired power forming the PV module. Then, the coefficients *N*_*P*_ and *N*_*S*_ are added to Eq. (2) of a solar cell, which are the number of modules in parallel and the number of solar cells in series, respectively. The current–voltage characteristic equation of a solar panel is shown in Eq. (6):

6 $$ I = N_{S} I_{L} - N_{P} I_{0} \left( {e^{{\frac{{q\left( {\frac{V}{{N_{S} }} + \frac{{IR_{S} }}{{N_{P} }}} \right)}}{{aKT_{C} }}}} - 1} \right) - \frac{{V\left( {\frac{{N_{P} }}{{N_{S} }}} \right) + IR_{S} }}{{R_{SH} }} $$

Equation (6) is reduced to Eq. (7), because shunt resistance does not affect the efficiency of a solar cell, since resistance tends to be very large or finite. Thus, *R*_*SH*_ = ∞ is assumed. However, the series resistance affects significantly the behaviour of the solar cell; therefore, for simple simulations the current provided by the solar panel described by Eq. (7):

7 $$ I = N_{S} I_{L} - N_{P} I_{0} \left( {e^{{\frac{{q\left( {\frac{V}{{N_{S} }} + \frac{{IR_{S} }}{{N_{P} }}} \right)}}{{aKT_{C} }}}} - 1} \right) $$

Once the equations that model the behaviour of PV modules have been developed, the main parameters and variables of the Mitsubishi Electric PV-TD185MF5 (185 Wp) PV module are presented in Tables 1 and 2, respectively.

**Table 1:** Model parameters.

Parameter	Definition	Value [units]
N_s_	Number of serial panels	50 [–]
N_p_	Number of parallel panels	1 [–]
*a*	Ideality factor	1.805 [–]
*q*	Electron’s charge	1.6 × 10^–19^ C
*K*	Boltzmann constant	1.38 × 10^–23^ J/°K
*Tc*_*Ref*_	Panel’s reference temperature	298 °K
*R*_*s*_	Series resistance	0.325 Ω
*R*_*sh*_	Shunt resistance	218.37 Ω
G_*Ref*_	Solar radiation at reference conditions	1,000 W/m^2^
$\alpha_{{I_{SC} }}$	Short-circuit current temperature coefficient	0.057%/°C
$V_{OC}$	Open circuit voltage	30.6 V
$I_{SC}$	Short circuit current	8.13 A
*E*_*G*_	Band gap energy	30.6 J
I_oRef_	Inverse saturation current at reference conditions	3.84 × 10^−10^ A

**Table 2:** Model variables.

Parameter	Definition	Units
$I$	Output current of the solar cell	A
*V*	Voltage generated in the solar cell	V
$I_{0}$	Inverse saturation current of the diode	A
$T_{C}$	Operating temperature of the solar cell	°C
$I_{L}$	Photo generated current	A
$I_{D}$	Current through the diode	A
$I_{SH}$	Resistance loss current in parallel	A

### PV module characterization: maximum power vs temperature

The MPPT algorithm proposed in the current study consists on a parametrization of an optimal current function *vs* maximum power. This linear function depends on the temperature. Subsequently, the optimal current function for each temperature has been calculated.

The procedure is the following one. Firstly, the control measures the current, the generated power and temperature of the panel. The temperature has been used as variable, since the irradiance is much more expensive to measure than the temperature. In fact, the temperature is a smoother variable than the irradiance, thus the control does not need to change the optimal current function so quickly.

Thereafter, an optimal current function by the MPPT algorithm has been parametrized. That is, a function created from a linear regression performed with the optimal points (MPP) at each relation power-current.

By using this optimal current linear function, the algorithm calculates the current set point. In that way, the control forces the PV module to work in the optimal current line. Taking into account the dynamic of PV module, the control always converges after several cycles to the optimal point. The stability of this MPPT algorithm has been verified in the next section in order to assure the convergence to this point.

**Figure 3:**
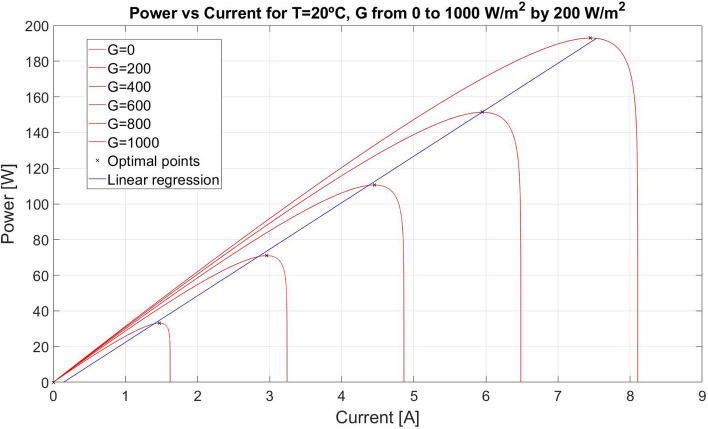
Maximum power vs optimal current for 20 °C.

Figure 3 shows different maximum power *vs* optimal current curves at 20 °C. Each curve is calculated for different irradiances with a constant temperature. The optimal points follow a line represented in blue colour in Fig. 3 and the coefficients of this line depend on temperature.

Thus, the blue line of Fig. 3 illustrates the MPP positions. Thereupon, if the control algorithm forces the PV module to work in this optimal line depending on temperature, the module dynamics converges to MPP.

The PV module output voltage depends on three variables: the module current (*I*), the irradiance (*G*) and the module temperature (*T*):

8 $$ V = f\left( {I,G,T} \right) $$

This output voltage allows defining the output power (*P*) of the module:

9 $$ P = V \cdot I = I \cdot f\left( {I,G,T} \right) $$

**Figure 4:**
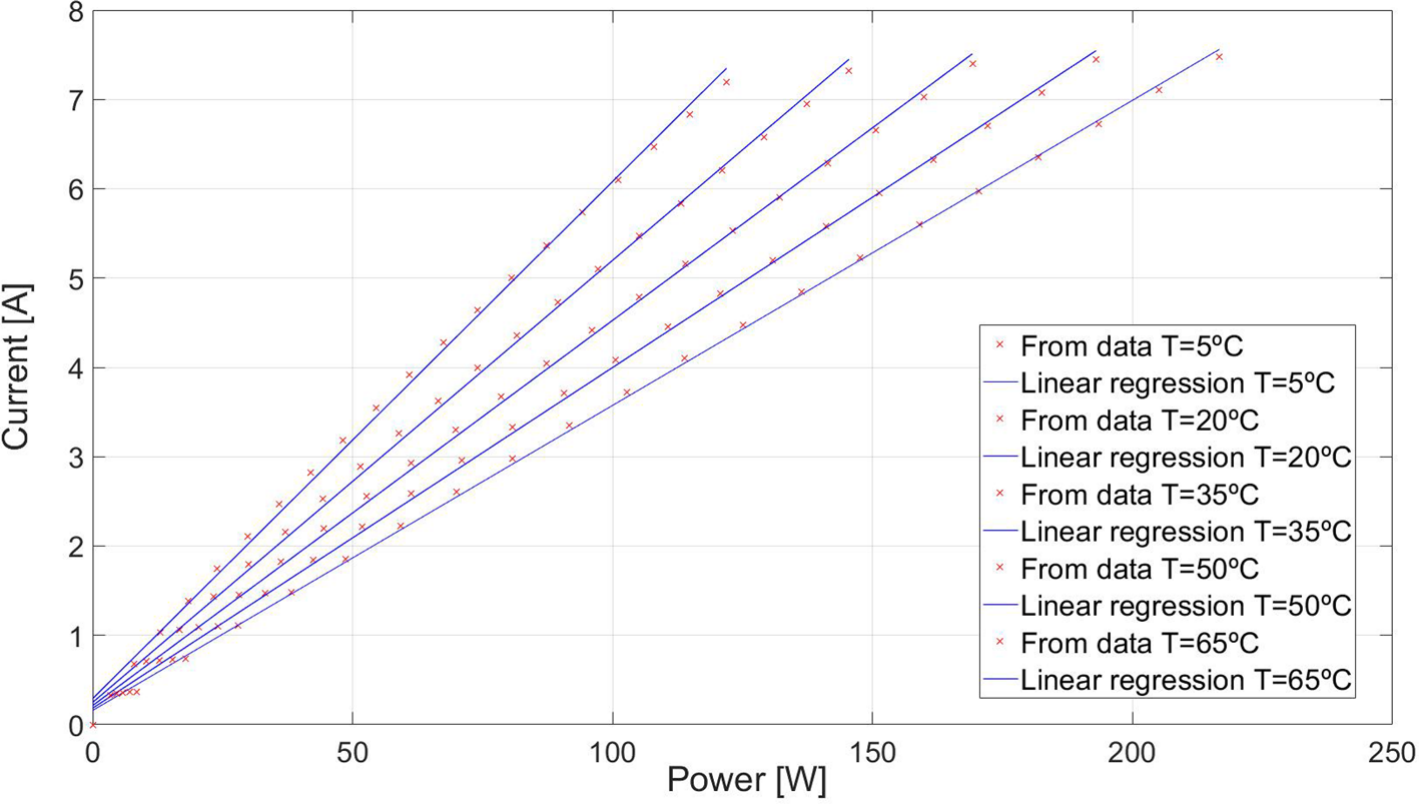
Maximum power vs optimal current curves for different temperatures.

Besides, the PV module maximum power (*P*_*max*_) values subject to a given temperature and irradiance can be calculated as shown in Fig. 4. The optimal current is a function of irradiance and temperature. If temperature is known, the maximum power and the optimal current depend on irradiance. Hence, power follows a curve that depends only on irradiance. Due to the optimal current dependence on irradiance, it can be defined a curve between maximum power and optimal current.

10 $$ P_{max} \left( {G,T} \right) = max_{s.t:G,T} \left( {P\left( {I,G,T} \right)} \right) $$

11 $$ I_{optimal} \left( {G,T} \right) = arg_{s.t:G,T} \left( {P\left( {I,G,T} \right)} \right) $$

Therefore, the real curve for a given temperature can be plotted. Finally, it can be stated that the optimal current for a given temperature for different irradiances follows a line. In order to make this affirmation the authors have proposed to eliminate the irradiance.

12 $$ I_{optimal} \left( {G,T} \right) = F\left( {P_{max} ,T} \right) $$

Hence, the MPPT algorithm presented in this paper must follow Eq. (12). If this equation is forced by the control, it can be said that the PV panel will operate in an optimal point.

Then a linear regression to specify the optimal current could be defined as in Eq. (13):

13 $$ I_{optimal} = a\left( T \right) \cdot P_{max} + b\left( T \right) $$

### Temperature based MPPT algorithm

The PV module dynamics is defined as a discrete time system, as the introduced MPPT algorithm works with $t_{k}$ sample time. Thus, the MPPT algorithm generates a current set point. The panel achieves quickly the current set point, since the power electronics has a relay control with hysteresis. This relay control has a negligible dynamics; consequently, it is considered that the module output current at *t*_*k*_ instant is approximately the current set point, see Eq. (2):

14 $$ P\left( {t_{k} } \right) = I\left( {t_{k} } \right) \cdot f\left( {I\left( {t_{k} } \right),G\left( {t_{k} } \right),T\left( {t_{k} } \right)} \right) $$

It is assumed that this control is able to measure the temperature and the obtained power. Therefore, following Eq. (13), the next sample time current $I\left( {t_{k + 1} } \right)$ is determined by Eq. (15):

15 $$ I\left( {t_{k + 1} } \right) = a\left( {T\left( {t_{k} } \right) \cdot P\left( {t_{k} } \right) + bT\left( {t_{k} } \right)} \right) $$

Equations (14) and (15) can be combined, resulting the equivalent system of Eq. (16):

16 $$ I\left( {t_{k + 1} } \right) = a\left( {T\left( {t_{k} } \right) \cdot \left[ {I\left( {t_{k} } \right) \cdot f\left( {I\left( {t_{k} } \right),G\left( {t_{k} } \right),T\left( {t_{k} } \right)} \right)} \right] + bT\left( {t_{k} } \right)} \right) $$

Consequently, the output current dynamics is summarized as follows in Eq. (17):

17 $$ I\left( {t_{k + 1} } \right) = F\left( {I\left( {t_{k} } \right),G\left( {t_{k} } \right),T\left( {t_{k} } \right)} \right) $$

**Figure 5:**
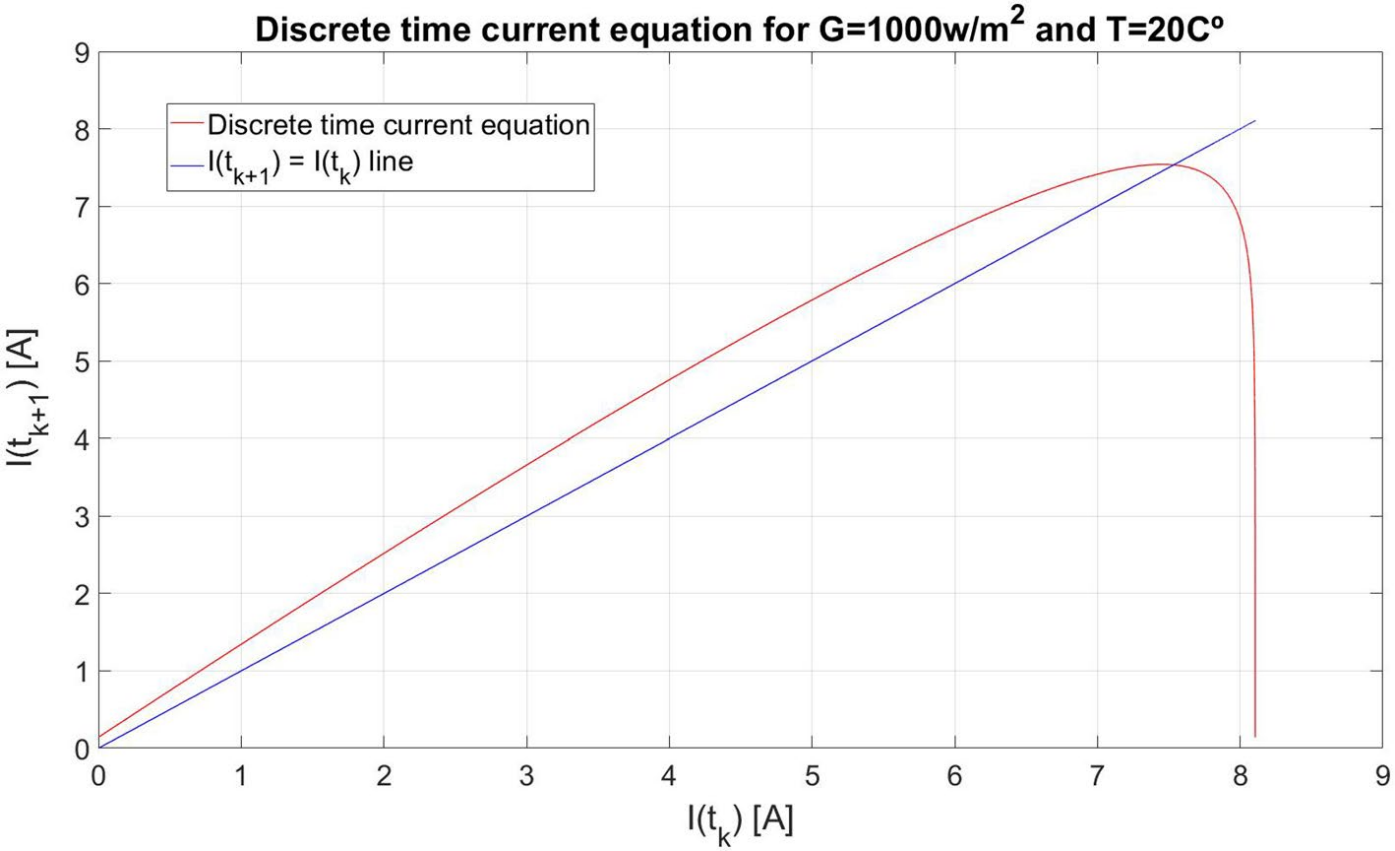
Discrete time current equation for 1000 W/m^2^ irradiance and 20 °C temperature.

Figure 5 shows the behaviour of this MPPT algorithm. The curve in red represents the dynamic function imposed by the MPPT, while the curve in blue illustrates the equilibrium line discussed in the following section.

### Temperature based MPPT algorithm stability analysis

The whole system follows a discrete time equation (see Eq. (16)). Discrete time current equation has an equilibrium point that is defined by the intersection point between Eq. (16) and the line $I\left( {t_{k + 1} } \right) = I\left( {t_{k} } \right)$. The discrete time current equation is in blue in Fig. 5.

The intersection point with line $I\left( {t_{k + 1} } \right) = I\left( {t_{k} } \right)$ is a point that fulfils Eq. (15). Subsequently, there is only one point in Eq. (15) for each irradiance. Hence, if the discrete time current equation tends to a point of Eq. (15), this equilibria point is the MPP for that temperature and that irradiance.

Current equation is a first order discrete time system, therefore this equation has one equilibria point, see Fig. 5. On the one hand, it is well known that if the discrete time current equation is above line $I\left( {t_{k + 1} } \right) = I\left( {t_{k} } \right)$ for *I*_*k*_ values less than the equilibria current *I*_*e*_, then the output current will increase. In the other hand, if discrete time equation is below line $I\left( {t_{k + 1} } \right) = I\left( {t_{k} } \right)$, for *I*_*k*_ values bigger than the equilibria current *I*_*e*_, then the output current will decrease, see Eqs. (18) and (19).

18 $$ I\left( {t_{k + 1} } \right) > I\left( {t_{k} } \right) if\,\, I\left( {t_{k} } \right) < I_{e} $$

19 $$ I\left( {t_{k + 1} } \right)\left\langle {I\left( {t_{k} } \right) if\,\, I\left( {t_{k} } \right)} \right\rangle I_{e} $$

This behaviour has been experimentally evaluated for all temperature levels and irradiance. Only various cases are shown in order to introduce the general problem. Despite Eqs. (18) and (19), the stability of equilibria point is not completely proven because a first order discrete time system is not able to make damped oscillations around the equilibria point.

In order to prove the stability of this equilibria point, a Lyapunov based function is proposed. The Lyapunov function has to fulfil with several mathematical conditions in order to be a valid energy function. These conditions are:

1. $$V\left( {I\left( {t_{k} } \right)} \right) > 0 if\,\, I\left( {t_{k} } \right) \ne I_{e}$$
2. $$V\left( {I\left( {t_{k} } \right) = I_{e} } \right) = 0 $$
3. $V\left( {I\left( {t_{k} } \right)} \right)$ must be continue for all $I\left( {t_{k} } \right)$

If $V\left( {I\left( {t_{k} } \right)} \right)$ is monotone negative respect to *k*, *I*_*e*_ will be stable.

20 $$ \Delta V = V\left( {I\left( {t_{k + 1} } \right)} \right) - V\left( {I\left( {t_{k} } \right)} \right) < 0 $$

The function proposed in this work is the following one:

$$ V\left( {I\left( {t_{k} } \right)} \right) = \left\{ {\begin{array}{*{20}l} {\left( {I\left( {t_{k} } \right) - I_{e} } \right)^{2} ,} \hfill & { if\,\, I\left( {t_{k} } \right) < I_{e} } \hfill \\ {0,} \hfill & {if\,\, I\left( {t_{k} } \right) = I_{e} } \hfill \\ {K_{v} \left( {I\left( {t_{k} } \right) - I_{e} } \right)^{2} , } \hfill & { if\,\, I\left( {t_{k} } \right) > I_{e} } \hfill \\ \end{array} } \right. $$

21 $$ K_{v} = \frac{{I_{e}^{2} }}{{\left( {I_{e} - I_{b} } \right)^{2} }} $$

where constant $K_{v}$ is defined in order to assure a negative difference in Lyapunov energy function. $I_{b}$ is a $I\left( {t_{k} } \right)$ current level such that $I\left( {t_{k + 1} } \right)$ is equal to a value such that $I\left( {t_{k + 1} } \right) + I\left( {t_{k} } \right) - 2I_{e}$ is equal to zero. This mathematical condition is necessary in order to assure that the proposed Lyapunov function is always positive, and its increment is always negative, see Eq. (20).

22 $$ I\left( {t_{k + 1} } \right) + I\left( {t_{k} } \right) - 2I_{e} = 0 $$

If Eqs. (22) and (17) are combined, the $I_{b}$ current definition is obtained.

23 $$ F\left( {I_{b} ,{\text{G}},{\text{T}}} \right) + I_{b} - 2I_{e} = 0 $$

The Lyapunov energy function increment is always negative for all $I\left( {t_{k} } \right)$ values. Different possible current spans are discussed in the following lines.

- If $I\left( {t_{k} } \right) < I_{e}$, it is important to notice that the next current it is always less than $I_{e}$. because $I_{e} > I\left( {t_{k + 1} } \right)$.
  The following inequalities are fulfilled, which are verified in Fig. 5.
  $$ \Delta V = \left( {I\left( {t_{k + 1} } \right) - I_{e} } \right)^{2} - \left( {I\left( {t_{k} } \right) - I_{e} } \right)^{2} = = I\left( {t_{k + 1} } \right)^{2} - I\left( {t_{k} } \right)^{2} - 2I_{e} \left( {I\left( {t_{k + 1} } \right) - I\left( {t_{k} } \right)} \right) $$
  Therefore, the Lyapunov functions increments are negatives for all $t_{k}$.
  $$ \Delta V = \left( {I\left( {t_{k + 1} } \right) - I\left( {t_{k} } \right)} \right)\left( {I\left( {t_{k + 1} } \right) + I\left( {t_{k} } \right) - 2I_{e} } \right) $$
- If $I\left( {t_{k} } \right) > I_{e}$, there are two different zones.
- The first zone is defined by this condition: $I_{e} \le I\left( {t_{k + 1} } \right)$. In this case, the Lyapunov energy function increment has an equivalent expression to Eq. (19).
  24 $$ \begin{gathered} \Delta V = K_{v} \left( {I\left( {t_{k + 1} } \right) - I_{e} } \right)^{2} - K_{v} \left( {I\left( {t_{k} } \right) - I_{e} } \right)^{2} \hfill \\ \Delta V = K_{v} \left( {I\left( {t_{k + 1} } \right) - I\left( {t_{k} } \right)} \right)\left( {I\left( {t_{k + 1} } \right) + I\left( {t_{k} } \right) - 2I_{e} } \right) \hfill \\ \end{gathered} $$
  In this zone the following inequality is fulfilled: $I\left( {t_{k + 1} } \right) < I\left( {t_{k} } \right)$.
  So $I_{e} \le I\left( {t_{k + 1} } \right) < I\left( {t_{k} } \right)$, and Lyapunov energy function increment is negative (see Eq. 24).
- In the second zone, when $I_{e} > I\left( {t_{k + 1} } \right)$ and $I\left( {t_{k} } \right) > I_{e}$, Lyapunov energy function increment has the following expression because $I\left( {t_{k + 1} } \right)$ is less than $I_{e}$.
  25 $$ \begin{gathered} \Delta V = \left( {I\left( {t_{k + 1} } \right) - I_{e} } \right)^{2} - K_{v} \left( {I\left( {t_{k} } \right) - I_{e} } \right)^{2} \hfill \\ \Delta V = \left( {I\left( {t_{k + 1} } \right) - \sqrt {K_{v} } I\left( {t_{k} } \right)} \right)\left[ {\left( {I\left( {t_{k + 1} } \right) + \sqrt {K_{v} } I\left( {t_{k} } \right)} \right)} \right] - 2I_{e} \left( {I\left( {t_{k + 1} } \right) - K_{v} I\left( {t_{k} } \right)} \right) + \left( {1 - K_{v} } \right)I_{e}^{2} \hfill \\ \end{gathered} $$

Taking into account Eq. (21), $\Delta V$ from Eq. (25) takes always, negative values.

**Figure 6:**
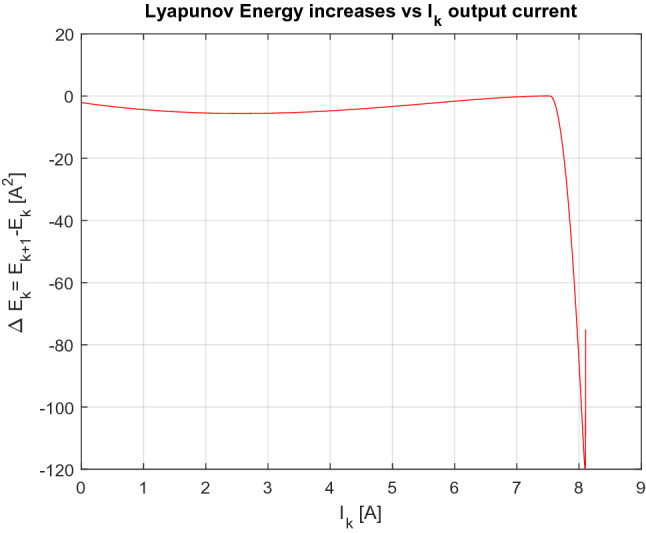
Lyapunov energy increments vs $I\left( {t_{k} } \right)$ for 1000 W/m^2^ irradiance and 20 °C temperature.

In Fig. 6, authors show Lyapunov energy function increment vs current $I\left( {t_{k} } \right)$. As it can be observed, all cases give negative values except to the equilibria point $I_{e}$.

## Conclusions

The current work presents an innovative method to improve the efficiency of PV systems, consisting on PV modules temperature based MPPT algorithm.

P&O and Incremental Conductance based algorithms move the operating point to approach the MPP, although the working conditions are constant. The main advantage of these conventional algorithms is that they are adaptive and robust almost on any type of photovoltaic modules. However, the main disadvantage consists on their oscillating behavior around the MPP. Therefore, a loss of energy is obtained when the environmental conditions (irradiance and temperature) are constant, since they move the operating point around the MPP but they do not reach accurately the MPP.

One of the advantages of the Lyapunov based algorithm developed in the current study compared to other algorithms is that only one input is required for the control algorithm: the PV module temperature. On the contrary, other algorithms such as P&O and Incremental Conductance need to use more than one input. Therefore, it makes our Lyapunov based algorithm easy to implement. Furthermore, this control law can be implemented into PV modules control system with a reasonably low cost.

The Lyapunov energy function based control algorithm presented in the current work forces the PV module to work along a linear function and in consequence, MPP is obtained in very few time steps. The number of cycles needed to achieve the optimal point are around 10, if the PV module starts in a very low current level (which is the worst case), as shown in Fig. 5. If the power electronics sample time is in the range between 10 to 1 ms, the time needed to achieve the optimal point is approximately from 100 to 10 ms. The stability of this MPPT algorithm has been determined in the present study via a Lyapunov energy function.

The most important drawback of this algorithm resides on the fact that an accurate PV model is required. Nevertheless, the PV module electronics needs to have an accurate model of PV in order to identify the dysfunctional states of PV modules.

As future work, the auto-tuning process of the photovoltaic panels should be developed. The PV modules used in the current work are well known by the authors; consequently, the MPPT algorithm presented in the current work is able to react very fast. However, in the case of using another type of PV modules, an algorithm to characterize immediately the necessary information required to adjust the MPPT is desirable. Moreover, additional research about how the MPPT based temperature control developed in the current work would behave in undesirable situations in photovoltaic panels such as partially shaded, failures or breakdowns is required. In addition, a PV module degradation study over time would be also interesting.
